# Engineering of *Burkholderia thailandensis* strain E264 serves as a chassis for expression of complex specialized metabolites

**DOI:** 10.3389/fmicb.2022.1073243

**Published:** 2022-11-17

**Authors:** Zong-Jie Wang, Xiaotong Liu, Haibo Zhou, Yang Liu, Lin Zhong, Xue Wang, Qiang Tu, Liujie Huo, Fu Yan, Lichuan Gu, Rolf Müller, Youming Zhang, Xiaoying Bian, Xiaokun Xu

**Affiliations:** ^1^Helmholtz International Lab for Anti-infectives, Shandong University-Helmholtz Institute of Biotechnology, State Key Laboratory of Microbial Technology, Shandong University, Qingdao, China; ^2^CAS Key Laboratory of Quantitative Engineering Biology, Shenzhen Institute of Synthetic Biology, Shenzhen Institute of Advanced Technology, Chinese Academy of Sciences, Shenzhen, China; ^3^Department of Microbial Natural Products, Helmholtz Institute for Pharmaceutical Research, Helmholtz Centre for Infection Research and Department of Pharmacy at Saarland University, Saarbrücken, Germany

**Keywords:** *Burkholderia*, chassis, heterologous expression, disorazol, rhizoxin

## Abstract

Heterologous expression is an indispensable approach to exploiting natural products from phylogenetically diverse microbial communities. In this study, we constructed a heterologous expression system based on strain *Burkholderia thailandensis* E264 by deleting efflux pump genes and screening constitutive strong promoters. The biosynthetic gene cluster (BGC) of disorazol from *Sorangium cellulosum* So ce12 was expressed successfully with this host, and the yield of its product, disorazol F_2_, rather than A_1_, was improved to 38.3 mg/L by promoter substitution and insertion. In addition to the disorazol gene cluster, the BGC of rhizoxin from *Burkholderia rhizoxinica* was also expressed efficiently, whereas no specific peak was detected when shuangdaolide BGC from *Streptomyces* sp. B59 was transformed into the host. This system provides another option to explore natural products from different phylogenetic taxa.

## Introduction

Natural products and their derivatives are important sources of new drugs (Newman and Cragg, 2020). With the development of next-generation sequencing technologies, an increasing amount of genomic data is available in public databases, and bioinformatic analysis has indicated that most of them are unexplored (Paoli et al., 2022). More than 99% of microbial organisms are currently unculturable (Pace, 1997); therefore, exploiting these treasures has attracted the interest of researchers worldwide. Heterologous expression has been confirmed as an effective strategy for obtaining natural products from slow-growing or even unculturable organisms, as well as poorly explored organisms, due to the lack of efficient genetic manipulation tools (Huo et al., 2019; Paoli et al., 2022).

**Figure 1:**
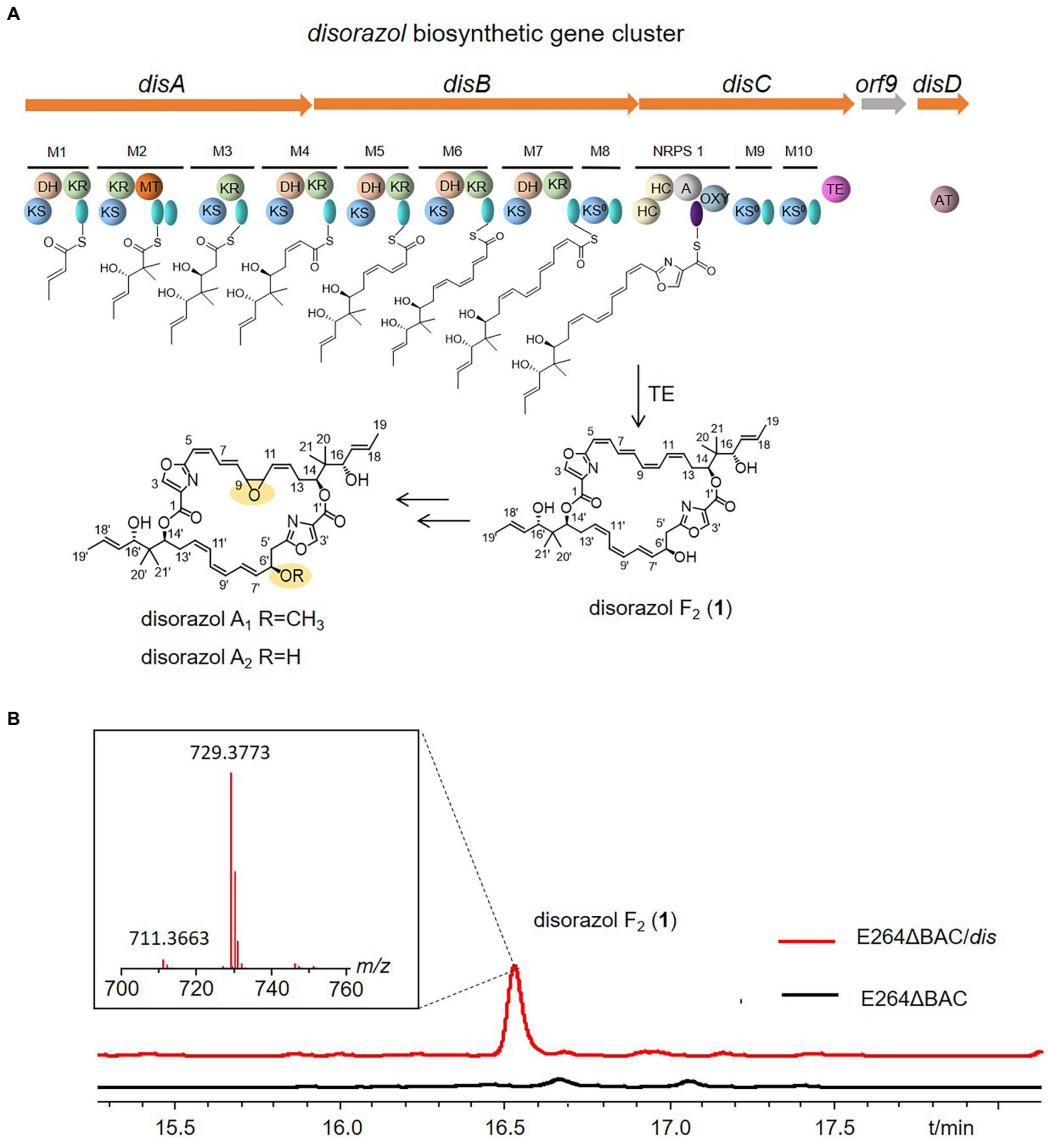
Domain organization of the disorazole biosynthetic gene cluster from *Sorangium cellulosum* So ce12 **(A)**, and disorazol F_2_ produced by E264ΔBAC/*dis* **(B)**. KS: ketosynthase, AT: acyl transferase, green oval: acyl carrier protein (ACP), HC: condensation/heterocyclization, A: adenylation, purple oval: peptidyl carrier protein (PCP), OXY: oxygenase, KR: ketoreductase, DH: dehydratase, MT: C-methyltransferase, TE: thioesterase,

Disorazols are a class of macrocyclic polyketides discovered in the myxobacterial strain *S. cellulosum* So ce12 (Jansen, 1994), and the flagship compound, disorazol A_1_, can inhibit the proliferation of different cancer cell lines at close to picomolar levels by interfering with microtubular dynamics (Elnakady et al., 2004; Menchon et al., 2018). The disorazol gene cluster was identified in So ce12 by transposon mutagenesis (Carvalho et al., 2005; Kopp et al., 2005). It is composed of four clustered genes (*disABCD*) and unidentified genes encoding tailoring enzymes responsible for epoxidation and methylation, which may lie outside of the biosynthetic gene cluster (BGC). Among them, *disABC* encode hybrid *trans*-AT PKS/NRPS megaenzymes, and *disD* encodes a separate protein that contains an acyl transferase domain and a possible oxidoreductase domain (Figure 1A). In our previous study, the disorazol BGC cloned in a bacterial artificial chromosome library was reconstructed by Red/ET-mediated recombineering (Zhang et al., 1998) and expressed in the model myxobacterium *Myxococcus xanthus* DK1622, considering the difficulties of culture and genetic manipulation of the original producer (Tu et al., 2016). However, this heterologous host grows relatively slowly and the yield remains quite low (<1 mg/L).

To overcome this problem, the strains from Burkholderiales with high GC content and abundant secondary metabolites (Kunakom and Eustáquio, 2019) can be used to express the disorazol gene cluster, which was inspired by the success of employing *Schlegelella brevitalea* DSM 7029 (Tang et al., 2019) as an efficient heterologous host to produce myxobacteria-derived epothilones, vioprolides and myxochelins (Bian et al., 2017; Yan et al., 2018; Liu J. et al., 2021). *Burkholderia thailandensis* E264, isolated from rice fields in Thailand, is a low virulence member of genus *Burkholderia* compared with *B. pseudomallei*, and *B. mallei*. Although a few infection cases caused by *B. thailandensis* in immunocompromised patients were reported (Brett et al., 1998; Glass et al., 2006; Chang et al., 2017), it is extensively studied as a model organism of the factors controlling virulence or as a producer of secondary metabolites, such as the metabolite bactobolin produced by a hybrid PKS-NRPS gene cluster (Duerkop et al., 2009; Seyedsayamdost et al., 2010), thailandamide biosynthesized through PKS (Nguyen et al., 2008), and the acyldepsitripeptide histone deacetylase inhibitor burkholdac catalyzed by NRPS (Biggins et al., 2011). Abundant secondary metabolites indicated that it could provide abundant substrates for expressing heterologous BGCs. In addition to possessing huge biosynthetic potential, strain E264 also has a fast growth rate, which makes it more suitable as a heterologous host (Mao et al., 2017; Liu et al., 2022). However, as a member of the genus *Burkholderia*, the intrinsic multidrug-resistance of the wild-type strain E264 makes genetic manipulation difficult, which partially results from the resistance-nodulation-division family efflux pumps (Biot et al., 2011, 2013).

In this study, we first deleted multidrug-resistance genes and obtained several antibiotic-sensitive mutants. The native potent promoters in strain E264 were screened by transcriptome analysis and evaluated *in vivo* by cloning them into a promoterless luciferase reporter vector. Selected promoters were used to improve the yields of disorazol in this heterologous host. Finally, the heterologous expression system was used to express the gene clusters from other phylogenetic taxa. The BGC of rhizoxin cloned by Red/ET-mediated direct cloning (Fu et al., 2012) was expressed successfully, whereas no specific peak was detected in the fermentation of the heterologous host containing shuangdaolide BGC cloned by direct cloning from *Streptomyces* sp. B59.

## Results

### Improved characteristics of engineered strain E264

During the course of expressing the disorazol gene cluster, *B. thailandensis* E264 showed better performance than the well-studied host DSM 7029 (Bian et al., 2017; Yan et al., 2018; Liu J. et al., 2021) in terms of growth rate and production. To facilitate genetic manipulation, three efflux pumps in strain E264, AmrAB-OprA (abbreviated as A), BpeAB-OprB (B), and BpeEF-OprC (C) (Biot et al., 2011, 2013), were inactivated by homologous recombination combined with *pheS* counterselection mediated seamless gene deletions (Barrett et al., 2008), and mutants with one, two, or three pump knockouts were obtained. Antibiotic susceptibility determined by the disc diffusion test was shown in tables S4 and S5. For most antibiotics, the mutant with one pump knockout (E264ΔA, E264ΔB, or E264ΔC) showed improved antibiotic sensitivity compared to the wild-type, suggesting that they are involved in multidrug resistance. Therefore, the mutant with three pump knockouts (E264ΔBAC) was constructed and used to express gene clusters or characterize promoters in subsequent experiments.

**Figure 2:**
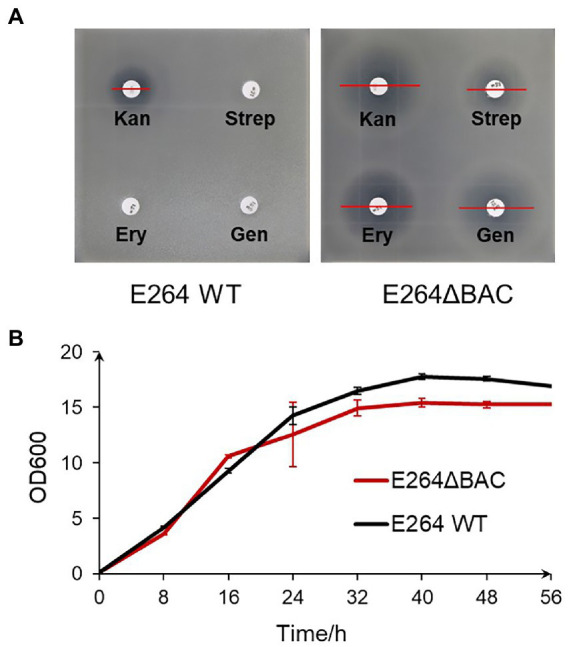
Characteristics of engineered strain E264. **(A)** Antibiotic sensibility test of mutant E264ΔBAC compared with wild-type strain E264. Kan, kanamycin 30 μg/disc; Strep, streptomycin 10 μg/disc; Ery, erythromycin 15 μg/disc; Gen, gentamicin 10 μg/disc. **(B)** Growth curves of both wild-type strain E264 and mutant E264ΔBAC.

Compared with the wild-type, E264ΔBAC was more sensitive to the tested antibiotics, except ampicillin (Figure 2A, Table 1). The minimum inhibitory concentrations (MIC) of the tested antibiotics against mutant E264ΔBAC were much lower than the concentrations used to select transformants (Supplementary Table S6). The MIC value (<7.5 μg/ml) of kanamycin against the mutant was much lower than the concentration (300 μg/ml) used to select transformants of the wild type (Kang et al., 2011). The MIC values of tetracycline, chloramphenicol, and erythromycin are lower than 0.6, 1.9, and 3.1 μg/ml, respectively. The antibiotic-sensitive mutant E264ΔBAC facilitates the genetic engineering of this potential heterologous host with low-concentration antibiotics as a selectable marker. In addition, the growth rate of the mutant E264ΔBAC was not affected by engineering, and the growth curve of the mutant was similar to that of the wild-type strain E264, except that the maximum OD600 was slightly lower (Figure 2B).

**Table 1:** The growth of mutant E264ΔBAC under different concentration of antibiotics.

Antibiotics	Concentration/μg‧ml^−1^					
Streptomycin	50	25	12.5	6.3	3.1	1.6
Gentamicin	10	5	2.5	1.3	0.6	
Tetracycline	10	5	2.5	1.3	0.6	
Apramycin	25	12.5	6.3	3.1	1.6	0.8
Kanamycin	30	15	7.5	3.8	1.9	0.9
Chloramphenicol	30	15	7.5	3.8	1.9	0.9
Erythromycin	25	12.5	6.3	3.1	1.6	0.8
Spectinomycin	100	50	25	12.5	6.3	3.1

0.05 < OD600 < 0.10.1 < OD600 < 0.50.5 < OD600 < 1.0OD600 > 1.The grey shade represents the OD600 in different concentration of antibiotic.

### Production of disorazol in E264ΔBAC

After obtaining the mutant, the plasmid p15A-dis (Supplementary Figure S1; Tu et al., 2016) was introduced into the mutant E264ΔBAC *via* conjugation mediated by the donor strain *Escherichia coli* WM3064 (Dehio and Meyer, 1997). A specific peak at *m/z* 729.4 was observed in the crude extract of the mutant with the disorazol gene cluster, and the area of the specific peak produced by mutant E264ΔBAC was much higher than that of *S. brevitalea* DSM 7029, which we used for heterologous expression of *cis*-PKS-derived epothilone (Figure 1B and Supplementary Figure S2; Bian et al., 2017), suggesting that E264 is more suitable for expressing this *trans-*AT PKS gene cluster. However, the molecular weight of the product (**1**) was different from that of disorazol A_1_ (Figure 1B). The molecular formula of **1** was predicted to be C_42_H_52_N_2_O_9_ based on HR-ESIMS (*m/z* 729.3773 [M + H]^+^, calcd 729.3746; Figure 1B), which is consistent with disorazol F_2_. Furthermore, the ^1^H and ^13^C NMR spectra of **1** confirmed that the product is disorazol F_2_ (**1**) (Supplementary Table S7, Supplementary Figures S3 and S4; Jansen, 1994). Compared to disorazol A_1_, the epoxide at C-9/C-10 in **1** was replaced by a double bond, and the hydroxyl group at C-6′ was not methylated (Figure 1A).

The heterologous expression of disorazol in a phylogenetically distant relative of the myxobacterial strain *S. cellulosum* could exclude the influence of homologous genes in the previously reported myxobacterial host *M. xanthus* DK1622 (Tu et al., 2016) and facilitate the elucidation of its biosynthetic pathway. The *orf9* encoding a ubiquinone biosynthesis protein Coq4 is not necessary for disorazol production (Tu et al., 2016). The product produced by the heterologous host *B. thailandensis* E264 is disorazol F_2_ rather than A_1_ or A_2_ produced by original producer So ce12 or myxobacterial host DK1622, indicating that the epoxidation and methylation of disorazols may be catalyzed by enzymes encoded by genes located outside of the gene cluster. Another debate regarding disorazols is the installation of a hydroxyl group at C-6. Theoretically, the DH7 domain could catalyze the elimination of hydroxyl groups at C-6 and C-6′ and form symmetrical macrodiolide products; however, most natural disorazols are unsymmetrical owing to the presence of the hydroxyl group at C-6′. The product obtained from E264ΔBAC::Ptet-*dis* is disorazol F_2_, an unsymmetrical macrodiolide, which suggests that the hydroxyl group at C-6′ is not installed during post-PKS modification.

### Yield improvement of disorazol F_2_

**Figure 3:**
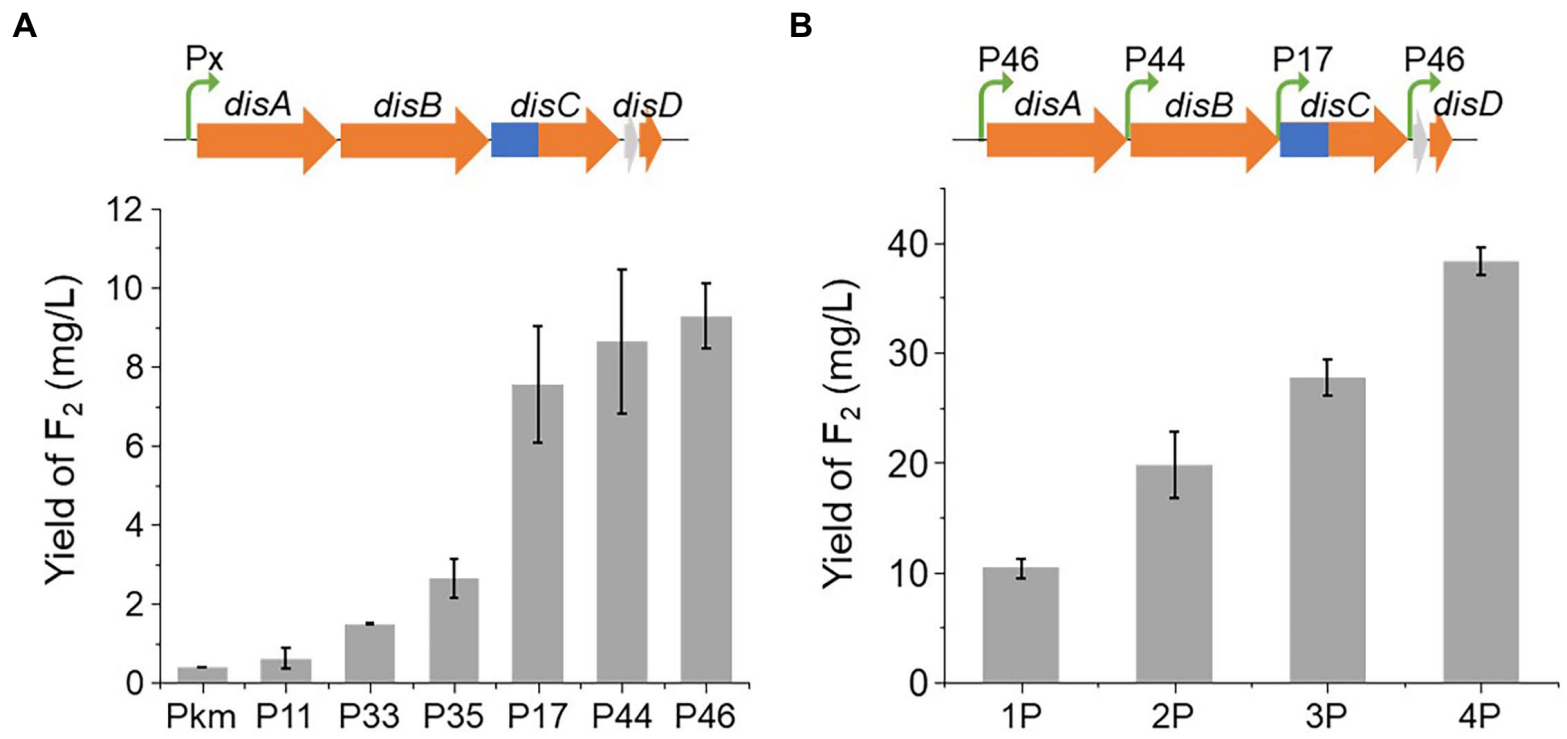
The yield of disorazol F_2_ produced by E264ΔBAC with different promoter strategies. **(A)** The yields of F_2_ (**1**) in E264ΔBAC::attB/dis from cluster expression promoted by different promoters (Px). **(B)** The yields of F_2_ (**1**) in E264ΔBAC::attB/dis driven by different numbers of promoters.

Initially, the yield of disorazol F_2_ (**1**) was less than 0.4 mg/L. To improve the yield of F_2_ (**1**), we first characterized potent native promoters in E264. Fifty native promoters with high expression levels at different phases of growth in M9 broth were selected based on the data of transcriptome analysis. However, the data of transcriptome may be affected by sample processing procedures, such as liquid nitrogen freezing, we further characterized the promoters *in vivo* by a luciferase assay to obtain a series of potent constitutive promoters of E264 (Supplementary Figure S5, Supplementary Table S8; Ouyang et al., 2020). To avoid possible positional effects, an *attB* site, the attachment site of site-specific recombination, was integrated into the locus of operon BpeEF-OprC, resulting in the mutant E264ΔBAC::*attB*. The potent promoters P11 (promoter of a hypothetical protein), P17 (promoter of a radical SAM protein), P33 (promoter of a hypothetical protein), P35 (promoter of a peroxiredoxin), P44 (promoter of a membrane protein), and P46 (promoter of a DUF4148 domain-containing protein) were employed to promote the expression of the disorazol gene cluster, resulting in a significant increase in yield (Figure 3A). Among them, the optimal promoter, P46, resulted in an approximately 23-fold increase in the average yield of disorazol F_2_ (**1**) to 9.3 mg/L. Promoters P17 and P44 showed similar effects, which led to an obvious increase in yield to 7.5 and 8.6 mg/L, respectively. Other promoters also improved the yields by 1.5-fold to 6.5-fold. To further improve the yield, promoters P46, P44, P17, and P46 were inserted upstream of genes *disA*, *disB*, *disC*, and *disD* using the RedEx method (Supplementary Figure S6; Song et al., 2020), which generated mutants 1P (P46), 2P (P46 + P44), 3P (P46 + P44 + P17), and 4P (P46 + P44 + P17 + P46; Figure 3B). The yield of disorazol F_2_ (**1**) showed a positive correlation with the number of promoters, which reached to 38.3 mg/L in mutant 4P-*dis*, 96-fold higher than that of E264ΔBAC::Ptet-*dis*. The significantly improved efficiency of heterologous expression encouraged us to express BGCs from other phylogenetical taxa.

### Expression of BGCs from other phylogenetical taxa

**Figure 4:**
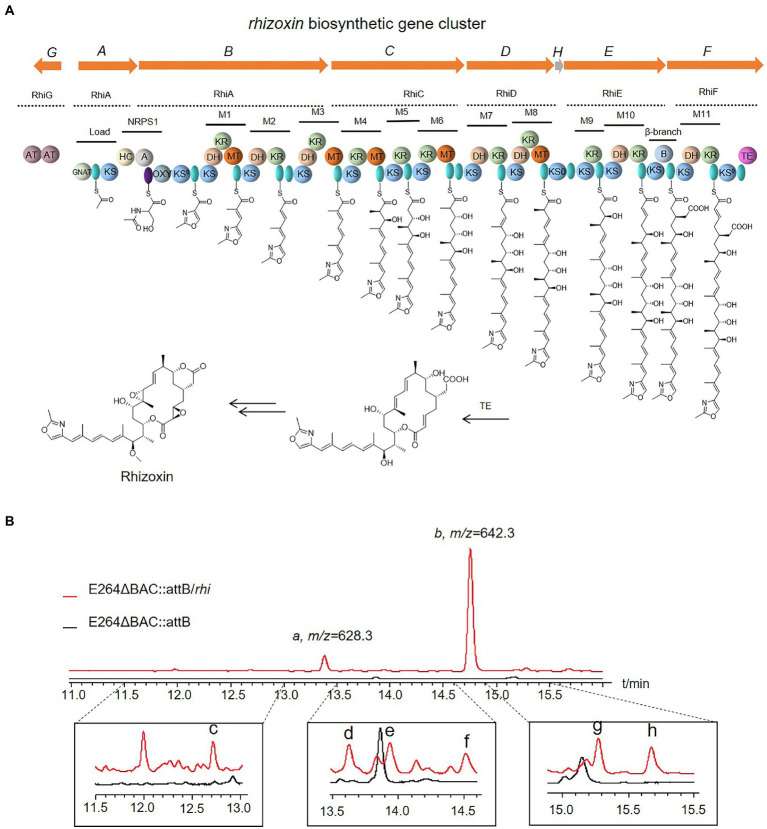
Organization of the rhizoxin (*rhi*) biosynthesis gene cluster and model for rhizoxin biosynthesis **(A)**, and the fermentation products of E264ΔBAC::attB/rhi **(B)**. GNAT: N-acetyltransferase; KS: ketosynthase, AT: acyl transferase, green oval: ACP, HC: condensation/heterocyclization, A: adenylation, purple oval: PCP, OXY: oxygenase, KR: ketoreductase, DH: dehydratase, MT: C-methyltransferase, TE: thioesterase, B: unknown domain possibly involved in β-branching.

In addition to the gene cluster from myxabacteria, the BGCs of rhizoxin (rhi) from *Burkholderia rhizoxinica* (Partida-Martinez and Hertweck, 2007) and shuangdaolide (sdl; Supplementary Figure S7) from *Streptomyces* sp. B59 (Liu Y. et al., 2021) were transferred to E264ΔBAC::*attB*. No specific peak was detected in the crude extract of E264ΔBAC::*attB*/sdl, whereas a series of specific peaks were observed in the fermentation product of E264ΔBAC::*attB*/rhi, whose molecular weights were consistent with the reported rhizoxins (Figure 4). Specific peaks a and b with a high proportion were detected at *m/z* 628.3 with *t*_R_ = 13.4 min and at *m/z* 642.3 with *t*_R_ = 14.8 min. Based on HRMS, the molecular formula of the compound in peak a was established as C_35_H_49_NO_9_ (*m/z* 628.3483 [M + H]^+^, calcd 628.3482), which is identical to that of rhizoxins M_1_, Z_2_, and S_2_; peak b may contain rhizoxin M_2_ or Z_2_ (C_36_H_51_NO_9_, *m/z* 642.3636 [M + H]^+^, calcd 642.3637). In addition to the major peaks, we also observed some specific peaks in a relatively low proportion of the fermentation product of E264ΔBAC::*attB*/rhi. Their MS/MS spectra were similar to peaks a and b, and HRMS suggested that they were also related to known rhizoxins (Supplementary Table S9). The BGC of rhizoxin from closely related species was expressed successfully in the host based on strain E264, and the potential of this heterologous expression system still needs to be further developed.

### Cytotoxicity test of disorazol F_2_ (**1**)

Owing to the low yield of the original producer So ce12, the cytotoxicity of disorazol F_2_ has never been reported in previous studies. In this study, the cytotoxicity of disorazol F_2_ (**1**) was evaluated against three human cancer cell lines (HepG2, HCT116, and A549; Table 2). Although one-two orders of magnitude weaker than epoxide disorazols A_1_ and A_2_ (Tu et al., 2016), disorazol F_2_ still exhibited potent activity against the tested cell lines with IC_50_ values ranging from 0.25 to 3.11 nM, one to three orders of magnitude stronger than clinically used doxorubicin or vincristine. The cytotoxicity assay compared with previously reported data (Irschik et al., 1995; Elnakady et al., 2004; Hopkins and Wipf, 2009; Tu et al., 2016) suggested that the activity of disorazols is affected by the epoxide at C-9/C-10, which suggests the tailoring enzyme of the disorazol pathway is still a significant task.

**Table 2:** Activity of disorazol F_2_ (**1**) against human cancer cell lines (IC_50_, nM).

Compounds	HepG2	HCT116	A549
F_2_ (**1**)	0.25 ± 0.01	3.11 ± 0.10	2.74 ± 0.56
Vincristine	6.62 ± 0.48	50.05 ± 3.68	44.28 ± 4.55
Doxorubicin	830 ± 119	793 ± 90	919 ± 35

IC_50_ values are calculated from three independent biological replicates.

## Discussion

Disorazols are a family of natural products with nanomolar cytotoxicity. However, the difficulties in culture and genetic manipulation of the original producer have limited further study. The heterologous expression system developed in this study efficiently overcomes these problems. This provides a powerful tool for illuminating this fantastic pathway. Heterologous expression in strain E264 indicated that the enzymes catalyzed epoxidation and methylation outside of the gene cluster. The gene cluster of disorazol also possesses some interesting characteristics unusual in classical PKS, such as a missing loading domain in module 1 and repeated ACP domains in module 2, as well as these ‘redundant’ domains in genes *disB* and *disC*. These interesting points may be elucidated in the near future by expressing domain (s) inactivation or deletion of the disorazol gene cluster with an efficient heterologous host.

This heterologous expression system also provides a platform for producing unnatural disorazol by PKS engineering. Natural products are designed to help producers occupy an ecological niche rather than to help people cure diseases. Therefore, it is effective to improve drug properties of them by increasing structural diversity (Floss, 2006). We believe that an increasing number of disorazol derivatives will be generated using this platform, which will promote drug development in this fantastic family.

Finally, myxobacteria are becoming an important source of natural products for drug discovery and still possess enormous biosynthetic potential (Weissman and Müller, 2010; Bader et al., 2020). The gene cluster of epothilone, a marketed anti-cancer drug isolated from *Sorangium cellulosum*, was successfully expressed in strain DSM 7029 with higher yields after multiple medium optimization and genome engineering (Bian et al., 2017; Yu et al., 2020). The heterologous expression system based on strain E264 provides another choice for the exploration of natural products from myxobacteria.

In addition to the advantages mentioned above, some problems must be addressed in this heterologous expression system. First, although strain E264 have been extensively studied in lab, as a class II opportunistic pathogen, potential risks need to be evaluated to ensure safety during large-scale fermentation. Additional measures, such as appropriate containment, are needed to ensure safety, which will significantly increase costs in industrial production. To overcome this problem, the construction of attenuated mutants through genetic engineering may be feasible. Secondly, this study obtained disorazol F_2_ rather than A_1_, which might leave the real “natural products” in the process of expressing other gene clusters. This problem needs to be addressed in other systems. Finally, the expression of shuangdaolide in the host failed, which may have resulted from their phylogenetic relationships. This result indicates that various hosts are required to express gene clusters from different taxa.

In summary, we developed an efficient heterologous expression system based on streamlined *B. thailandensis* E264, in which the disorazol gene cluster was expressed successfully. The yield of the heterologous product, disorazol F_2_, was improved 96-fold using promoter substitution and insertion. The expression of the rhizoxin gene cluster from *Burkholderia rhizoxina* was also successful, whereas no specific peak was detected in the fermentation of the host containing the shuangdaolide gene cluster. Furthermore, the safety and compatibility of the heterologous expression system should be improved.

## Materials and methods

### Bacterial strains

*Burkholderia thailandensis* E264 was purchased from German collection of microorganisms and cell cultures GmbH (DSMZ) and was cultured in low-salt Luria-Bertani medium (1% tryptone, 0.5% yeast extract, 0.1% NaCl) at 37°C. The markerless mutants of strain E264 were established as described in section construction of deletion mutants. *Escherichia coli* GB05-red was used for mediating homologous recombination between a linear and circular DNA molecular (LCHR) while *E. coli* GB05-dir mediating linear plus linear recombination (LLHR). *Escherichia coli* GB05-dir-gyrA462 or GB05-red-gyrA462 were used for constructing plasmids with counterselectable marker CcdB. *Escherichia coli* WM3064 was employed as a conjugal donor (Dehio and Meyer, 1997). Unless otherwise specified, the strains of *Escherichia coli* were cultured under the same conditions with strain E264. Tetracycline (Tet) was added to a final concentration of 50 μg/ml for selective growth of wild type strain of *B. thailandensis* E264. For mutant E264ΔBAC, antibiotics were added to final concentrations of 30 μg/ml kanamycin (Kan) or 5 μg/ml gentamicin (Genta) as required, for strains of *E. coli*, the concentrations were 30 μg/ml Kan, 100 μg/ml ampicillin (Amp) or 15 μg/ml chloramphenicol (Cm).

### Construction of deletion mutants

The genomic DNA (gDNA) of strain E264 was isolated using phenol – chloroform – isoamyl alcohol mixture (Wang et al., 2020). To obtain markerless mutants, genetic tools developed by Barrett *et al* were modified and applied in this study (Barrett et al., 2008). Briefly, about 1 kb regions upstream and downstream of targeted genes amplified by PCR using PrimeSTAR® HS DNA polymerase with GC buffer were cloned into vector pBR322-amp-tet-pheS (linearized with restriction enzymes *Xab*I and *Nco*I) by strain GB05-dir. The plasmids with homology arms were introduced into strain E264 by natural transformation (Garcia, 2017), and correct clones verified by colony PCR were cultured in LSLB without antibiotic for 12 h to eliminate selection marker. The overnight cultures were centrifuged and washed twice with liquid M9G medium (M9 medium of Sangon, Shanghai supplemented with 20 mM Glucose), then 50 μl cell suspensions were plated on M9G plates supplementing with 0.1% 4-Chloro-D, L-phenylalanine (Sigma-Aldrich) to screen the recombinants with selection marker deletion. Markerless mutants were further verified by colony PCR. To generate mutant E264ΔBAC::*attB*, the sequence of *attB* (GGGTGCCAGGGCGTGCCCTTGGGCTCCCCGGGCGCGTA) was inserted between the homology arms of pBR322-Amp-Tet-pheS-oprC through oligonucleotide synthesis, and the plasmid was introduced into mutant E264ΔBA followed by selection and counterselection successively.

### Antibiotic sensitivity test

To make test plates, the overnight cultures (200 μl) of strain E264 or E264ΔBAC were mixed with 50 ml melted LSLB agar medium. Then, the paper discs containing different antibiotics (HangweiTM, Hangzhou) were placed on the media. The plates were cultured at 37°C overnight to measure the diameter of inhibition zone.

### Plasmids construction for promoter characterization

The amp-ccdB cassette flanked with two restriction enzyme *Nde*I sites and about 40 bp homology arms was amplified from plasmid p15A-amp-ccdB using primers amp-ccdB-P11-F/R (Wang et al., 2018), the PCR product and plasmid p15A-genta-int-attP-P11-firefly (Ouyang et al., 2020) were electroporated into *E. coli* GB05-red-gyrA462 expressed recombinases Redα/Redβ, which resulted in plasmid p15A-genta-int-attP-amp-ccdB-firefly. Colonies growth on plates supplemented with Amp and Genta were transferred into 1.8 ml fresh LB broth with appropriate antibiotics and incubated at 37°C overnight. The plasmid DNA was extracted from the overnight culture and confirmed by restriction analysis.

Promoter Px was amplified using primers PX-F/R with 40 bp homology arms, and plasmid p15A-genta-int-attP-amp-ccdB-firefly was linearized with restriction endocuclease *Nde*I. The products of PCR and restriction enzyme digestion were electroporated into *E. coli* GB05-dir expressed recombinases RecE/RecT, which resulted in plasmid p15A-genta-int-attP-Px-firefly.

### Luciferase assay

Plasmids with different promoters located upstream of luciferase gene were introduced into E264ΔBAC::*attB* by conjugation mediated by WM3064 (Dehio and Meyer, 1997), The expression levels of report gene in transformants were detected by Luciferase Assay System of Promega Corporation. Briefly, 20 μl cell lysates prepared according the instruction were mixed with 100 μl of Luciferase Assay Reagent, and the light produced by the mixture was quantified by GloMaxTM 96 Microplate Luminometer of Promega Corporation.

### Reconstruction of disorazol gene cluster

Cloning vector BAC-cm flanked with *Pac*I/*Hpa*I sites and homology arms was amplified by PCR from plasmid pBeloBAC11 (digested with *Bam*HI and *Hin*dIII) using primers bac-dis-F/R. The products of PCR and plasmid p15A-dis (Tu et al., 2016) linearized with *Xba*I were electroporated into *E. coli* GB05-dir, which resulted in plasmid pBAC-cm-dis.

Plasmid p15A-genta-int-attP-Px-firefly was linearized with *Mfe*I, then cassette p15A-genta-int-attP-Px with homology arms was amplified by PCR using primers Px-dis-F/R. The PCR product and linearized plasmid pBAC-cm-dis (digested with *Pac*I and *Hpa*I) were assembled by LLHR mediated by GB05-dir, which resulted in plasmid p15A-genta-int-attP-Px-dis.

To construct plasmids with multiple promoters and improve the yield of disorazol, the strategy of RedEx was employed (Song et al., 2020). Breifly, plasmid p15A-genta-int-attP-P46-dis was electroporated into *E. coli* GB05-red-gyrA462, then the cassette 2P-amp-ccdB, dis (17985–18044)-*Pac*I-amp-ccdB-*Pac*I-dis (18015–18044)-P44-dis (18053–18110; the first nucleotide of *disA* was designated as 1), generated by fusion PCR was electroporated into *E. coli* GB-red-gyrA462 with plasmid p15A-genta-int-attP-P46-dis. The cassette 2P-amp-ccdB was inserted upstream of *disB* through LCHR mediated by recombinases Redα/Redβ which resulted in plasmid p15A-genta-int-attP-2P-dis-amp-ccdB. The plasmid p15A-genta-int-attP-2P-dis-amp-ccdB was digested with *Pme*I, and 100 ng linearized plasmid DNA was treated with 0.2 U T4pol (New England BioLabs) in a 20 μl reaction at 25°C for 30 min, 75°C for 20 min, 50°C for 20 min and then held at 4°C in a thermocycler. The reaction mixture was electroporated into *E. coli* GB2005 cells after desalting treatment. The recombinant p15A-genta-int-attP-2P-dis (2 promoters) with promoter P44 inserting upstream *disB* was identified with *Kpn*I/*Pme*I restriction analysis.

Utilizing the strategy of RedEX, promoter P17 was inserted upstream of gene *disC* and P46 was inserted upstream of gene *disD* successively, which resulted in plasmid p15A-genta-int-attP-3P-dis (3 promoters) and p15A-genta-int-attP-4P-dis (4 promoters), respectively.

### Metabolite extraction, HPLC, and LC–MS analyses

Single colonies were inoculated into 2 ml Eppendorf tubes containing 1.8 ml LB broth supplemented with appropriate antibiotics and incubated at 37°C overnight with shaking at 900 rpm. The seed cultures (1%, v/v) were transferred into 50 ml M9 broth in 250 ml Erlenmeyer flask and incubated at 30°C with shaking at 200 rpm for 24 h before 2% XAD-16 resin was added, then the incubation was continued for another 48 h. The resin was collected by centrifugation and resuspended with 50 ml of methanol. The mixtures were shaken at 30°C, 200 rpm for 2 h. The methanol was removed by evaporation *in vacuo*, and residues were dissolved with 1 ml of methanol. After filtering with 0.22 μm membrane, the crude extracts were analyzed by UPLC-MS (UltiMate 3,000 UPLC system combined with Bruker amazon SL Ion Trap mass spectrometer). The C18 column (2.1 × 100 mm. 2.2 μm, Thermo) was utilized to analyze the crude extracts at a 0.3 ml/min flow rate using the following program: 0–3 min 5% solvent B; 3–22 min, 5 95% with linear gradient; then, 22–25 min, 5% solvent B (Solvent A, Milli Q water supplemented with 0.1% formic acid; Solvent B, acetonitrile supplemented with 0.1% formic acid). Mass spectra were acquired in positive ion mode.

### Isolation of disorazol F_2_ (**1**)

The crude extract from 10 L culture of E264ΔBAC::Ptet/*dis* was fractionated with Sephadex LH-20 column (GE Healthcare) chromatography using MeOH as a mobile phase. Fractions containing F_2_ (**1**) were combined and further purified by semipreparative reverse-phase HPLC (Agilent ZORBAX SB-C18 column, 250 × 9.4 mm, 5 μm; gradient elution 0–2 min, 64% ACN; 2–17 min, 64–90% ACN; 17–22 min, 95% ACN, 22–25 min, 64% ACN; 2.5 ml/min). Finally, 1.5 mg of disorazol F_2_ (**1**) was obtained with retention time at 15.1 min.

### Quantitative analysis of disorazol F_2_ (**1**)

The standard curve of disorazol F_2_ (**1**) was established by measuring the area of absorption spectrum at UV 280 nm containing 0.05, 0.1, 0.2, 0.5 and 1.0 mg/ml disorazol F_2_ (**1**). The yield of disorazol F_2_ (**1**) was determined by reference to the standard curve.

## Data availability statement

The datasets presented in this study can be found in online repositories. The accession number for the RNA-Seq data in this paper is GEO: PRJNA889572.

## Author contributions

XX, XB, YZ, and Z-JW: designed research. XX, Z-JW and XL: performed research. HZ, YL, LZ, XW, QT, LH, FY, LG, RM, XB, and YZ: analyzed data. Z-JW, XX, HZ, and XB: wrote the paper. All authors contributed to the article and approved the submitted version.

## Funding

This work was supported by the National Key R&D Program of China (2021YFC2100500, 2019YFA0905700, 2019YFA09004000), the National Natural Science Foundation of China (32161133013, 32070060, 32100052, and 32170038), the Shandong Provincial Natural Science Foundation of China (ZR2019JQ11, ZR2019ZD18, and ZR2019ZD30), the 111 project (B16030), Youth Interdisciplinary Innovative Research Group of SDU (2020QNQT009), and the China Postdoctoral Science Foundation (2020M682169).

## Conflict of interest

The authors declare that the research was conducted in the absence of any commercial or financial relationships that could be construed as a potential conflict of interest.

## Publisher’s note

All claims expressed in this article are solely those of the authors and do not necessarily represent those of their affiliated organizations, or those of the publisher, the editors and the reviewers. Any product that may be evaluated in this article, or claim that may be made by its manufacturer, is not guaranteed or endorsed by the publisher.

## References

[ref1] Bader C. D. Panter F. Müller R. (2020). In depth natural product discovery—myxobacterial strains that provided multiple secondary metabolites. Biotechnol. Adv. 39:107480. doi: 10.1016/j.biotechadv.2019.107480, PMID: 31707075

[ref2] Barrett A. R. Kang Y. Inamasu K. S. Son M. S. Vukovich J. M. Hoang T. T. (2008). Genetic tools for allelic replacement in *Burkholderia* species. Appl. Environ. Microbiol. 74, 4498–4508. doi: 10.1128/AEM.00531-08, PMID: 18502918 PMC2493169

[ref3] Bian X. Tang B. Yu Y. Tu Q. Gross F. Wang H.. (2017). Heterologous production and yield improvement of epothilones in Burkholderiales strain DSM 7029. ACS Chem. Biol. 12, 1805–1812. doi: 10.1021/acschembio.7b00097, PMID: 28467833

[ref4] Biggins J. B. Gleber C. D. Brady S. F. (2011). Acyldepsipeptide HDAC inhibitor production induced in *Burkholderia thailandensis*. Org. Lett. 13, 1536–1539. doi: 10.1021/ol200225v, PMID: 21348454 PMC3103185

[ref5] Biot F. V. Lopez M. M. Poyot T. Neulat-Ripoll F. Lignon S. Caclard A.. (2013). Interplay between three RND efflux pumps in doxycycline-selected strains of *Burkholderia thailandensis*. PLoS One 8:e84068. doi: 10.1371/journal.pone.0084068, PMID: 24386333 PMC3873969

[ref6] Biot F. V. Valade E. Garnotel E. Chevalier J. Villard C. Thibault F. M.. (2011). Involvement of the efflux pumps in chloramphenicol selected strains of *Burkholderia thailandensis*: proteomic and mechanistic evidence. PLoS One 6:e16892. doi: 10.1371/journal.pone.0016892, PMID: 21347382 PMC3036723

[ref7] Brett P. J. Deshazer D. Woods D. E. (1998). *Burkholderia thailandensis* sp. nov., a *Burkholderia pseudomallei*-like species. Int. J. Syst. Bacteriol. 48 Pt 1, 317–320. doi: 10.1099/00207713-48-1-317, PMID: 9542103

[ref8] Carvalho R. Reid R. Viswanathan N. Gramajo H. Julien B. (2005). The biosynthetic genes for disorazoles, potent cytotoxic compounds that disrupt microtubule formation. Gene 359, 91–98. doi: 10.1016/j.gene.2005.06.003, PMID: 16084035

[ref9] Chang K. Luo J. Xu H. Li M. Zhang F. Li J.. (2017). Human infection with *Burkholderia thailandensis*, China, 2013. Emerg. Infect. Dis. 23, 1416–1418. doi: 10.3201/eid2308.170048, PMID: 28726626 PMC5547772

[ref10] Dehio C. Meyer M. (1997). Maintenance of broad-host-range incompatibility group P and group Q plasmids and transposition of Tn5 in *Bartonella henselae* following conjugal plasmid transfer from *Escherichia coli*. J. bacteriol. 179, 538–540. doi: 10.1128/jb.179.2.538-540.1997, PMID: 8990308 PMC178726

[ref11] Duerkop B. A. Varga J. Chandler J. R. Peterson S. B. Herman J. P. Churchill M. E. A.. (2009). Quorum-sensing control of antibiotic synthesis in *Burkholderia thailandensis*. J. Bacteriol. 191, 3909–3918. doi: 10.1128/JB.00200-09, PMID: 19376863 PMC2698390

[ref12] Elnakady Y. A. Sasse F. Lunsdorf H. Reichenbach H. (2004). Disorazol A1, a highly effective antimitotic agent acting on tubulin polymerization and inducing apoptosis in mammalian cells. Biochem. Pharmacol. 67, 927–935. doi: 10.1016/j.bcp.2003.10.029, PMID: 15104246

[ref13] Floss H. G. (2006). Combinatorial biosynthesis-potential and problems. J. Biotechnol. 124, 242–257. doi: 10.1016/j.jbiotec.2005.12.001, PMID: 16414140 PMC1865499

[ref14] Fu J. Bian X. Hu S. Wang H. Huang F. Seibert P. M.. (2012). Full-length rec E enhances linear-linear homologous recombination and facilitates direct cloning for bioprospecting. Nat. Biotechnol. 30, 440–446. doi: 10.1038/nbt.2183, PMID: 22544021

[ref15] Garcia E. (2017). *Burkholderia thailandensis*: genetic manipulation. Curr. Protoc. Microbiol. 45:4C.2.1-4C.2.15. doi: 10.1002/cpmc.27, PMID: 28510362 PMC5434709

[ref16] Glass M. B. Gee J. E. Steigerwalt A. G. Cavuoti D. Barton T. Hardy R. D.. (2006). Pneumonia and septicemia caused by *Burkholderia thailandensis* in the United States. J. Clin. Microbio. 44, 4601–4604. doi: 10.1128/jcm.01585-06, PMID: 17050819 PMC1698378

[ref17] Hopkins C. D. Wipf P. (2009). Isolation, biology and chemistry of the disorazoles: new anti-cancer macrodiolides. Nat. Prod. Rep. 26, 585–601. doi: 10.1039/b813799b, PMID: 19387496 PMC2711774

[ref18] Huo L. Hug J. J. Fu C. Bian X. Zhang Y. Müller R. (2019). Heterologous expression of bacterial natural product biosynthetic pathways. Nat. Prod. Rep. 36, 1412–1436. doi: 10.1039/c8np00091c 30620035

[ref19] Irschik H. Jansen R. Gerth K. Hofle G. Reichenbach H. (1995). Disorazol a, an efficient inhibitor of eukaryotic organisms isolated from myxobacteria. J. Antibiot. (Tokyo) 48, 31–35. doi: 10.7164/antibiotics.48.31, PMID: 7868386

[ref20] Jansen R. (1994). Disorazoles, highly cytotoxic metabolites from the sorangicin-producing bacterium *Sorangium cellulosum*, strain So ce 12. Liebigs. Ann. Chem. 1994, 759–773. doi: 10.1002/jlac.199419940802

[ref21] Kang Y. Norris M. H. Wilcox B. A. Tuanyok A. Keim P. S. Hoang T. T. (2011). Knockout and pullout recombineering for naturally transformable *Burkholderia thailandensis* and *Burkholderia pseudomallei*. Nat. Protoc. 6, 1085–1104. doi: 10.1038/nprot.2011.346, PMID: 21738123 PMC3564556

[ref22] Kopp M. Irschik H. Pradella S. Müller R. (2005). Production of the tubulin destabilizer disorazol in *Sorangium cellulosum*: biosynthetic machinery and regulatory genes. Chembiochem 6, 1277–1286. doi: 10.1002/cbic.200400459, PMID: 15892181

[ref23] Kunakom S. Eustáquio A. S. (2019). Burkholderia as a source of natural products. J. Nat. Prod. 82, 2018–2037. doi: 10.1021/acs.jnatprod.8b01068, PMID: 31294966 PMC6871192

[ref24] Liu J. Wang X. Dai G. Zhang Y. Bian X. (2022). Microbial chassis engineering drives heterologous production of complex secondary metabolites. Biotechnol. Adv. 59:107966. doi: 10.1016/j.biotechadv.2022.107966, PMID: 35487394

[ref25] Liu Y. Zhou H. Shen Q. Dai G. Yan F. Li X.. (2021). Discovery of polycyclic macrolide shuangdaolides by heterologous expression of a cryptic *trans*-AT PKS gene cluster. Org. Lett. 23, 6967–6971. doi: 10.1021/acs.orglett.1c02589, PMID: 34388000

[ref26] Liu J. Zhou H. Yang Z. Wang X. Chen H. Zhong L.. (2021). Rational construction of genome-reduced *Burkholderiales chassis* facilitates efficient heterologous production of natural products from proteobacteria. Nat. Commun. 12:4347. doi: 10.1038/s41467-021-24645-0, PMID: 34301933 PMC8302735

[ref27] Mao D. Bushin L. B. Moon K. Wu Y. Seyedsayamdost M. R. (2017). Discovery of scm R as a global regulator of secondary metabolism and virulence in *Burkholderia thailandensis* E264. Proc. Natl. Acad. Sci. U. S. A. 114, E2920–e2928. doi: 10.1073/pnas.1619529114, PMID: 28320949 PMC5389298

[ref28] Menchon G. Prota A. E. Lucena-Agell D. Bucher P. Jansen R. Irschik H.. (2018). A fluorescence anisotropy assay to discover and characterize ligands targeting the maytansine site of tubulin. Nat. Commun. 9:2106. doi: 10.1038/s41467-018-04535-8, PMID: 29844393 PMC5974090

[ref29] Newman D. J. Cragg G. M. (2020). Natural products as sources of new drugs over the nearly four decades from 01/1981 to 09/2019. J. Nat. Prod. 83, 770–803. doi: 10.1021/acs.jnatprod.9b01285, PMID: 32162523

[ref30] Nguyen T. Ishida K. Jenke-Kodama H. Dittmann E. Gurgui C. Hochmuth T.. (2008). Exploiting the mosaic structure of trans-acyltransferase polyketide synthases for natural product discovery and pathway dissection. Nat. Biotechnol. 26, 225–233. doi: 10.1038/nbt1379, PMID: 18223641

[ref31] Ouyang Q. Wang X. Zhang N. Zhong L. Liu J. Ding X.. (2020). Promoter screening facilitates heterologous production of complex secondary metabolites in Burkholderiales strains. ACS Synth. Biol. 9, 457–460. doi: 10.1021/acssynbio.9b00459, PMID: 31999442

[ref32] Pace N. R. (1997). A molecular view of microbial diversity and the biosphere. Science 276, 734–740. doi: 10.1126/science.276.5313.734, PMID: 9115194

[ref33] Paoli L. Ruscheweyh H.-J. Forneris C. C. Hubrich F. Kautsar S. Bhushan A.. (2022). Biosynthetic potential of the global ocean microbiome. Nature 607, 111–118. doi: 10.1038/s41586-022-04862-3, PMID: 35732736 PMC9259500

[ref34] Partida-Martinez L. P. Hertweck C. (2007). A gene cluster encoding rhizoxin biosynthesis in “*Burkholderia rhizoxina*”, the bacterial endosymbiont of the fungus *Rhizopus microsporus*. Chembiochem. 8, 41–45. doi: 10.1002/cbic.200600393, PMID: 17154220

[ref35] Seyedsayamdost M. R. Chandler J. R. Blodgett J. A. V. Lima P. S. Duerkop B. A. Oinuma K.-I.. (2010). Quorum-sensing-regulated bactobolin production by *Burkholderia thailandensis* E264. Org. Lett. 12, 716–719. doi: 10.1021/ol902751x, PMID: 20095633 PMC2821070

[ref36] Song C. Luan J. Li R. Jiang C. Wang H. (2020). RedEx: a method for seamless DNA insertion and deletion in large multimodular polyketide synthase gene clusters. Nucleic Acids Res. 48:e130. doi: 10.1093/nar/gkaa956, PMID: 33119745 PMC7736807

[ref37] Tang B. Yu Y. Liang J. Zhang Y. Bian X. Zhi X.. (2019). Reclassification of '*Polyangium brachysporum*' DSM 7029 as *Schlegelella brevitalea* sp. nov. Int. J. Syst. Evol. Microbiol. 69, 2877–2883. doi: 10.1099/ijsem.0.003571, PMID: 31274403

[ref38] Tu Q. Herrmann J. Hu S. Raju R. Bian X. Zhang Y.. (2016). Genetic engineering and heterologous expression of the disorazol biosynthetic gene cluster via red/ET recombineering. Sci. Rep-UK 6:21066. doi: 10.1038/srep21066, PMID: 26875499 PMC4753468

[ref39] Wang H. Li Z. Jia R. Yin J. Li A. Xia L.. (2018). ExoCET: exonuclease *in vitro* assembly combined with RecET recombination for highly efficient direct DNA cloning from complex genomes. Nucleic Acids Res. 46:e28. doi: 10.1093/nar/gkx1249, PMID: 29240926 PMC5861427

[ref40] Wang Z. J. Zhou H. Zhong G. Huo L. Tang Y. J. Zhang Y.. (2020). Genome mining and biosynthesis of primary amine-acylated desferrioxamines in a marine gliding bacterium. Org. Lett. 22, 939–943. doi: 10.1021/acs.orglett.9b04490, PMID: 31994894

[ref41] Weissman K. J. Müller R. (2010). Myxobacterial secondary metabolites: bioactivities and modes-of-action. Nat. Prod. Rep. 27, 1276–1295. doi: 10.1039/c001260m, PMID: 20520915

[ref42] Yan F. Auerbach D. Chai Y. Keller L. Tu Q. Hüttel S.. (2018). Biosynthesis and heterologous production of vioprolides: rational biosynthetic engineering and unprecedented 4-methylazetidinecarboxylic acid formation. Angew. Chem. Int. Ed. Engl. 57, 8754–8759. doi: 10.1002/anie.201802479, PMID: 29694699

[ref43] Yu Y. Wang H. Tang B. Liang J. Zhang L. Wang H.. (2020). Reassembly of the biosynthetic gene cluster enables high epothilone yield in engineered *Schlegelella brevitalea*. ACS Syn. Bio. 9, 2009–2022. doi: 10.1021/acssynbio.0c00100, PMID: 32603592

[ref44] Zhang Y. Buchholz F. Muyrers J. P. Stewart A. F. (1998). A new logic for DNA engineering using recombination in *Escherichia coli*. Nat. Genet. 20, 123–128. doi: 10.1038/2417, PMID: 9771703

