# Supplementary material for: Engineering of Burkholderia thailandensis strain E264 serves as a chassis for expression of complex specialized metabolites
Source: Front Microbiol. 2022 Nov 17;13:1073243. doi: 10.3389/fmicb.2022.1073243 (PMC9712229; doi:10.3389/fmicb.2022.1073243)
Supplement: Supplementary file 1 [file Data_Sheet_1.docx]

Supplementary Material

**Contents**

Table S1. Plasmids used in this study

Table S2. Strains used in this study

Table S3. Primers used in this study

Table S4. The comparison of resistance between wild type E264 and mutants with one pump knockout

Table S5. The comparison of resistance between wild type E264 and mutants with two pumps knockout

Table S6. The comparison of resistance between E264 wild type and mutant E264ΔBAC (μg/mL)

Table S7. The ^1^H (600 MHz) and ^13^C NMR (150 MHz) Data of disorazol F_2_ (**1**) in MeOH-*d*_4_

Table S8. Selected 50 promoter regions from *B. thailandensis* E264

Table S9. HRMS data of E264ΔBAC::attB/rhi fermentation products

Figure S1. Plasmid map of p15A-dis

Figure S2. Heterologous expression of disorazol gene cluster in different hosts

Figure S3. The ^1^H NMR spectrum of disorazol F_2_ (**1**) in MeOH-*d*_4_

Figure S4. The ^13^C NMR spectrum of disorazol F_2_ (**1**) in MeOH-*d*_4_

Figure S5. The promoter strength evaluated by firefly luciferase assay

Figure S6. Seamless insertion of promoter P44

Figure S7. Proposed biosynthesis of shuangdaolides

# Supplementary Tables

## Supplementary Table S1. Plasmids used in this study

| **Name** | **Description** | **Ref.** |
| --- | --- | --- |
| pBR322-amp-tet-pheS-oprA | Plasmid used to destroy operon *AmrAB-OprA* of E264 | This study |
| pBR322-amp-tet-pheS-oprB | Plasmid used to destroy operon *BpeAB-OprB* of E264 |  |
| pBR322-amp-tet-pheS-oprC | Plasmid used to destroy operon *BpeEF-OprC* of E264 |  |
| pBR322-amp-tet-pheS-oprC-*attB* | Plasmid used to destroy operon *BpeEF-OprC* of E264 and insert *attB* sequence into corresponding locus |  |
| p15A-genta-int-*attP*-P11-firefly | p15A replicon, genta*^R^*, an origin of transfer (oriT), *attP* site and phiC31 integrase, firefly luciferase under the control of P11 promoter from DSM 7029 | (1) |
| BAC-cm-*dis* | Disorazol gene cluster with BAC replicon and *cm^R^* | This study |
| p15A-genta-int-*attP*-amp-ccdB-firefly | P11(DSM 7029) promoter replaced by the amp-ccdB cassette | This study |
| p15A-genta-int-*attP*-P_Tn5-km_-firefly | firefly luciferase promoted by PTn5-km promoter | This study |
| p15A-genta-int-*attP*-P_tet_-firefly | firefly luciferase promoted by Ptet promoter | This study |
| p15A-genta-int-*attP*-P_X_-firefly | firefly luciferase promoted by Px (Px: one of selected promoters from E264) | This study |
| p15A-genta-int-*attP*-Px-*dis* | *dis* promoted by Px (Px: one of selected promoters from E264) | This study |
| p15A-genta-int-*attP*-1P-*dis* | *dis* promoted by promoter P46 | This study |
| p15A-genta-int-*attP*-2P-*dis* | *disA* promoted by P46, *disB* was promoted by P44 | This study |
| p15A-genta-int-*attP*-3P-*dis* | *disA* promoted by P46, *disB* promoted by P44, *disC* promoted by P17 | This study |
| p15A-genta-int-*attP*-4P-*dis* | *disA* promoted by P46, *disB* promoted by P44, *disC* promoted by P17, *disD* promoted by P46 | This study |

## Supplementary Table S2. Strains used in this study

| **Strain** | **Description** | **Ref.** |
| --- | --- | --- |
| ***Escherichia coli*** |  |  |
| GB2005 | F-*mcr*A Δ(*mrr*-*hsd*RMS-*mcr*BC) *φ*80*lac*ZΔM15 Δ*lac*X74 *rec*A1 *end*A1 *ara*D139 Δ(*ara, leu*)7697 *gal*U *gal*K λ *rpsL nup*G*fhu*A::IS2 *rec*ET *redα*, phage T1-resistent | (2) |
| GB05-dir | GB2005, *ara*C-BAD-ETγA | (2) |
| GB05-red | GB2005, *ara*C-BAD-γβαA | (2) |
| WM3064 | *Escherichia coli* donor strain used for conjugation | (3) |
| ***Burkholderia thailandensis* E264** |  | (4) |
| E264ΔBAC | AmrAB-OprA, BpeAB-OprB and BpeEF-OprC knockout mutant | This study |
| E264ΔBAC::*attB* | E264ΔBAC mutant with *attB* site integrated | This study |
| E264ΔBAC::*attB*/*dis* | Disorazol gene cluster integrated into the *attB* of E264ΔBAC::*attB* | This study |
| E264ΔBAC::Ptet-*dis* | Disorazol gene cluster with tet promotor integrated onto the genome of E264ΔBAC by transposition | This study |
| E264ΔBAC::*attB*/P11-*dis* | p15A-genta-int-*attP*-P11-*dis* integrated to *attB* site of E264ΔBAC::*attB* | This study |
| E264ΔBAC::*attB*/P17-*dis* | p15A-genta-int-*attP*-P17-*dis* integrated to *attB* site of E264ΔBAC::*attB* | This study |
| E264ΔBAC::*attB*/P33-*dis* | p15A-genta-int-*attP*-P33-*dis* integrated to *attB* site of E264ΔBAC::*attB* | This study |
| E264ΔBAC::*attB*/P35-*dis* | p15A-genta-int-*attP*-P35-*dis* integrated to *attB* site of E264ΔBAC::*attB* | This study |
| E264ΔBAC::*attB*/P44-*dis* | p15A-genta-int-*attP*-P44-*dis* integrated to *attB* site of E264ΔBAC::*attB* | This study |
| E264ΔBAC::*attB*/P46-*dis* | p15A-genta-int-*attP*-P46-*dis* integrated to *attB* site of E264ΔBAC::*attB* | This study |
| E264ΔBAC::*attB*/1P-*dis* | E264ΔBAC/P46-*dis* | This study |
| E264ΔBAC::*attB*/2P-*dis* | p15A-genta-int-*attP*-2P-*dis* integrated to *attB* site of E264ΔBAC::*attB* | This study |
| E264ΔBAC::*attB*/3P-*dis* | p15A-genta-int-*attP*-3P-*dis* integrated to *attB* site of E264ΔBAC::*attB* | This study |
| E264ΔBAC::*attB*/4P-*dis* | p15A-genta-int-*attP*-4P-*dis* integrated to *attB* site of E264ΔBAC::*attB* | This study |

## Supplementary Table S3. Primers used in this study

| **Primers** | **Primer sequences (5'-3')** | **Application** |
| --- | --- | --- |
| OprA-*Xba*I-UpHA-F | AATAGGGGTTCCGCGCACATTTCCCCGAAAAGCATGCCTTAAG**TCTAGA**CGCTTCACGCTCCGGCATGC | To construct plasmid pBR322-amp-tet-phes-oprA |
| OprA-UpHA-R | CCGACCGGCCCCAAATTCGTCCCGGTCGAGCGACTTGCGAGGAAAACGTCTTCGCGGGCGGACGCAATC |  |
| OprA-DownHA-F | CGTTTTCCTCGCAAGTCGCTC |  |
| OprA-*Nco*I-DownHA-R | ACCGCATTAAAGCTTATCGATGATAAGCTGTCAAACATGAGA**CCATGG**CAAAGCGCTCGATGCGCGCTT |  |
| OprB-*Xba*I-UpHA-F | ACAAATAGGGGTTCCGCGCACATTTCCCCGAAAAGCATGCCTTAAG**TCTAGA**GCGTGAATAATAAGGGTTGCATC | To construct plasmid pBR322-amp-tet-phes-oprB |
| OprB-UpHA-R | GCTGCGCGTGCTCGTTGCGCTCGAGCGCCGCGATCTGCTGATCGTACGTTTGCGTGGCCACCGCGTTGT |  |
| OprB-DownHA-F | ACGTACGATCAGCAGATCGCG |  |
| OprB-*Nco*I-DownHA-R | ATAAACTACCGCATTAAAGCTTATCGATGATAAGCTGTCAAACATGAGA**CCATGG**CGCGCGGGCGATCGAACAGG |  |
| OprC-*Xba*I-UpHA-F | AATAGGGGTTCCGCGCACATTTCCCCGAAAAGCATGCCTTAAG**TCTAGA**ATCGGCGCGCAAGGCGAAAC | To construct plasmid pBR322-amp-tet-phes-oprC or pBR322-amp-tet-phes-oprC-*attB* |
| OprC-UpHA-R | TTGGGGAAACGACGTGGCCG |  |
| OprC-DownHA-F | GTCCTGCTTGTTCGGCTCGCGCGAACCGACGGCCACGTCGTTTCCCCAAAGCGCCGCATGCGACACGTC |  |
| OprC-attB-DownHA-F | CGGCCACGTCGTTTCCCCAA*GGGTGCCAGGGCGTGCCCTTGGGCTCCCCGGGCGCGTA*AGCGCCGCATGCGACACGTC |  |
| OprC-*Nco*I-DownHA-R | CCGCATTAAAGCTTATCGATGATAAGCTGTCAAACATGAGA**CCATGG**ATCTCGCAATGGACGCGCGACG |  |
| BAC-cm-F | TCGTACCCTTGGAGGCCCATGGCTGCTCGAGACAGCCGAC**ACGTTAAC**CACGCTGATAGTCTGATCGACA | To construct pBAC-cm-*dis* |
| BAC-cm-R | TTAGGAATTAATCATCTGGCCATTCGATGGTGTCGGGTCATGTGAGC**TTAATTAA**AGGGCACCAATAACTGCCTTAA |  |
| amp-ccdB-P11-F | CCGAACAGGCTTATGTCAACTGGGTTCGTGCCTTC**CATATG**CTAAATACATTCAAATATGTATCC | Construct p15A-genta-int-*attP*-amp-ccdB-firefly |
| amp-ccdB-P11-R | CGGGCCTTTCTTTATGTTTTTGGCGTCTTCCAT**CATATG**TGGGTTATATTCCCCAGAAC |  |
| Px-F | GCTTATGTCAACTGGGTTCGTGCCTTC-about 20 bp of Px | Construct p15A-genta-int-*attP*-Px-firefly (Px represent fifty E264 promoter) |
| Px-R | TTTCTTTATGTTTTTGGCGTCTTCCAT-about 20 bp of Px |  |
| P46-dis-F | TCGTACCCTTGGAGGCCCATGGCTGCTCGAGACAGCCGACGTTTTCGTTCCACTGAGATCTTAAGG | Construct p15A-genta-int-*attP*-P46-*dis* |
| P46-dis-R | ACGCCGATGATCGCAATGGCGTCCTGCTCCATGATTGACCTCCGGAATGTTGTATTTGC |  |
| P11-dis-R | ACGCTACGCCGATGATCGCAATGGCGTCCTGCTCCATGGATTCCTCCTTCGTCGTAGTC | With P46-*dis*-F to construct p15A-genta-int-*attP*-P11-*dis* |
| P17-dis-R | CACGCTACGCCGATGATCGCAATGGCGTCCTGCTCCATGGCGCATTCCCCTCGATATGG | With P46-dis-F to construct p15A-genta-int-*attP*-P17-*dis* |
| P33-dis-R | GGCACGCTACGCCGATGATCGCAATGGCGTCCTGCTCCATCAGCATTCTCCCGGCGGGG | With P46-*dis*-F to construct p15A-genta-int-*attP*-P33-*dis* |
| P35-dis-R | CTACGCCGATGATCGCAATGGCGTCCTGCTCCATGTTGCAGCTCCTTGAACTTGCATGG | With P46*dis*-F to construct p15A-genta-int-*attP*-P35-*dis* |
| P44-dis-R | ACGCCGATGATCGCAATGGCGTCCTGCTCCATATTTCTCCTCTCGAAATTGAGATTACC | With P46-*dis*-F to construct p15A-genta-int-*attP*-P44-*dis* |
| 2P-amp-ccdB-F | CGCTGCGCCGCTCGGCTATTACCAATCGACCTGGACCAGAAGCGCGCTTTGAACGTCGGG**GTTTAAAC**TAAATACATTCAAATATGTATC | Construct 2P-amp-ccdB cassette |
| 2P -amp-ccdB-3 | GCCCCGACGTTCAAAGCGCGCTTCTGGTCCAG**GTTTAAAC**AGCCCGCTCATTAGGCGGG |  |
| 2P-amp-ccdB-5 | GTTTAAACCTGGACCAGAAGCGCGCTTTGAACGTCGGGGCGGCAAGCGGTGTTGCACTC |  |
| 2P-amp-ccdB-R | ACGAGGATGGTGTCTGTGTGGCGGAGAGTGCTGGACATATTTCTCCTCTCGAAATTGAG |  |
| 3P-amp-ccdB-F | GGCGTACCGGGCGAGGAGCTGACTCGGCTCTACGCCATCCTGCAAGAGGAATGA**GTTTAAAC**TAAATACATTCAAATATGTATCC | Construct 3P-amp-ccdB cassette |
| 3P-amp-ccdB-3 | CGTCATTCCTCTTGCAGGATGGCGTAGAGCCG**GTTTAAAC**AGCCCGCTCATTAGGCGGG |  |
| 3P-amp-ccdB-5 | TGTTTAAACCGGCTCTACGCCATCCTGCAAGAGGAATGACGTGTAATGCCTGAAACCGTC |  |
| 3P-amp-ccdB-R | GGCAAACTCCTGGATGGTCATCGCGCTTTCCATCGTCATGCGCATTCCCCTCGATATGG |  |
| 4P-amp-ccdB-F | AGCCCCCGGCGCTCCACCAGGTGGTCGCGCACGTCCGCGAGGCGCTTTCATGA**GTTTAAAC**TAAATACATTCAAATATG | Construct 4P-amp-ccdB cassette |
| 4P-amp-ccdB-3 | AAGCCGGTCATGAAAGCGCCTCGCGGACGTGCGCGAC**GTTTAAAC**AGCCCGCTCATTAG |  |
| 4P-amp-ccdB-5 | TTAAACGTCGCGCACGTCCGCGAGGCGCTTTCATGACCGGCTTTCGGTCGAAATTAAC |  |
| 4P-amp-ccdB-R | GGGAGCTCCTCGTGGATGTCCAGGTGTCGCGCCATCGATGTCATTTGACCTCCGGAATGTTGTATTTG |  |

The homology arms are underlined and restriction enzyme sites are in bold.

## Supplementary Table S4. The comparison of resistance between wild type E264 and mutants with one pump knockout*^a, b^*

| Antibiotics | Diameter of inhibitory zone/mm | | | |
| --- | --- | --- | --- | --- |
|  | E264 | E264Δ*oprA* | E264Δ*oprB* | E264Δ*oprC* |
| Spectinomycin (100) | 17.4 ± 1.1 | 39.4 ± 0.8 | 29.0 ± 0.4 | 21.2 ± 0.9 |
| Norfloxacin (10) | 15.5 ± 1.2 | 20.0 ± 0.4 | 19.0 ± 0.5 | 18.3 ± 0.5 |
| Neomycin (30) | 11.3 ± 0.5 | 19.0 ± 0.9 | 11.6 ± 0.7 | 10.9 ± 0.3 |
| Gentamicin H (120) | 6.0 ± 0.0 | 22.2 ± 1.3 | 12.2 ± 0.7 | 10.4 ± 0.6 |
| Chloramphenicol (30) | 24.7 ± 1.0 | 23.6 ± 1.9 | 32.1 ± 1.2 | 27.7± 0.9 |
| Erythromycin (15) | 6.0 ± 0.0 | 22.8 ± 0.7 | 6.6 ± 0.1 | 6.7 ± 0.2 |
| Tobramycin (10) | 8.0 ± 0.5 | 18.0 ± 0.8 | 11.7 ± 0.7 | 11.8 ± 0.6 |
| Kanamycin (30) | 11.9 ± 0.7 | 26.1 ± 1.4 | 16.5 ± 0.7 | 19.2 ± 0.6 |
| Sulfonamides (300) | 6.0 ± 0.0 | 6.0 ± 0.0 | 11.3 ± 0.5 | 6.0 ± 0.0 |
| Tetracyclines (30) | 10.6 ± 0.3 | 17.8 ± 0.4 | 12.8 ± 0.6 | 13.0 ± 0.8 |
| Streptomycin (10) | 6.0 ± 0.0 | 16.9 ± 0.6 | 6.0 ± 0.0 | 6.0 ± 0.0 |
| Gentamicin (10) | 6.0 ± 0.0 | 18.9 ± 0.6 | 6.0 ± 0.0 | 6.0 ± 0.0 |

*^a^* ug/dics; *^b^* The diameter of dics is 6 mm. Three independent biological replicates were performed.

## Supplementary Table S5. The comparison of resistance between wild type E264 and mutants with two pumps knockout*^a, b^*

| Antibiotics | Diameter of inhibitory loop/mm | | | |
| --- | --- | --- | --- | --- |
|  | E264 | E264  Δ*oprA*Δ*oprB* | E264  Δ*oprA*Δ*oprC* | E264  Δ*oprB*Δ*oprC* |
| Spectinomycin (100) | 17.4 ± 1.1 | 32.5 ± 1.3 | 27.6 ± 0.8 | 22.0 ± 0.5 |
| Norfloxacin (10) | 15.5 ± 1.2 | 25.2 ± 0.5 | 17.9 ± 0.4 | 18.4 ± 0.4 |
| Neomycin (30) | 11.3 ± 0.5 | 20.2 ± 0.6 | 18.7 ± 0.6 | 14.5 ± 0.4 |
| Gentamicin H (120) | 6.0 ± 0.0 | 22.2 ± 0.8 | 19.6 ± 0.5 | 6.0 ± 0.0 |
| Chloramphenicol (30) | 24.7 ± 1.0 | 25.5 ± 1.0 | 24.6 ± 0.4 | 26.3 ± 0.8 |
| Erythromycin (15) | 6.0 ± 0.0 | 20.4 ± 0.4 | 23.6 ± 0.5 | 6.0 ± 0.0 |
| Tobramycin (10) | 8.0 ± 0.5 | 17.7 ± 0.7 | 16.5 ± 0.5 | 10.3 ± 0.5 |
| Kanamycin (30) | 11.9 ± 0.7 | 16.3 ± 0.6 | 22.0 ± 0.8 | 14.5 ± 0.3 |
| Sulfonamides (300) | 6.0 ± 0.0 | 13.0 ± 0.4 | 6.0 ± 0.0 | 11.8 ± 0.4 |
| Tetracyclines (30) | 10.6 ± 0.3 | 22.1 ± 0.7 | 18.9 ± 0.4 | 12.4 ± 0.5 |
| Streptomycin (10) | 6.0 ± 0.0 | 17.4 ± 0.4 | 18.3 ± 0.7 | 6.0 ± 0.0 |
| Gentamicin (10) | 6.0 ± 0.0 | 17.8 ± 0.4 | 19.4 ± 0.3 | 6.0 ± 0.0 |

*^a^* ug/dics; *^b^* The diameter of dics is 6 mm. Three independent biological replicates were performed.

## Supplementary Table S6. The comparison of resistance between E264 wild type and mutant E264ΔBAC (μg/mL)

|  | **Km** | **Strep** | **Em** | **Apra** | **Genta** | **Spect** | **Tet** | **Cm** | **Amp** |
| --- | --- | --- | --- | --- | --- | --- | --- | --- | --- |
| **E264** | 180 (+) | 300 (+) | 100 (+) | 100 (+) | 20 (+) | 300 (+) | 25 (+) | 90 (±) | 300 (+) |
| **E264ΔBAC** | 30 (-) | 50 (-) | 25 (-) | 25 (-) | 10 (-) | 100 (-) | 10 (-) | 30 (-) | 300 (+) |

+: Growth, -: No Growth, ±: Sporadic growth

**Supplementary Table S7. The ^1^H (600 MHz) and ^13^C NMR (150 MHz) Data of disorazol F_2_ (1) in MeOH-*d*_4_**

| Position | *δ***_C_** | *δ*_H_ (*J* in Hz) | Position | *δ***_C_** | *δ*_H_ (*J* in Hz) |
| --- | --- | --- | --- | --- | --- |
| 1 | 160.80 |  | 1ʹ | 160.65 |  |
| 2 | 132.88 |  | 2ʹ | 132.33 |  |
| 3 | 144.65 | 8.29 s | 3ʹ | 145.31 | 8.33 s |
| 4 | 162.74 |  | 4ʹ | 162.15 |  |
| 5 | 111.31 | 6.08 d (11.6) | 5ʹ | 36.73 | 2.86 m |
| 6 | 137.18 | 6.50 m | 6ʹ | 68.96 | 4.32 q (5.6) |
| 7 | 128.62 | 7.14 dd (12.2, 14.3) | 7ʹ | 135.12 | 5.83 dd (5.8, 14.9) |
| 8 | 135.39 | 6.80 dd (12.1, 14.3) | 8ʹ | 126.54 | 6.5 m |
| 9 | 128.90 | 6.02 m | 9ʹ | 128.62 | 6.02 m |
| 10 | 128.85 | 6.50 m | 10ʹ | 124.24 | 6.34 m |
| 11 | 126.71 | 6.50 m | 11ʹ | 127.73 | 6.50 m |
| 12 | 130.81 | 5.48 m | 12ʹ | 128.50 | 5.61 m |
| 13 | 27.97 | 2.86 m 2.34 m | 13ʹ | 27.86 | 2.86 m 2.34 m |
| 14 | 77.21 | 5.27 dd (1.8, 11.2) | 14ʹ | 77.07 | 5.30 dd (2.4, 11.5) |
| 15 | 41.24 |  | 15ʹ | 41.21 |  |
| 16 | 76.46 | 3.87 d (1.9) | 16ʹ | 76.41 | 3.86 d (2.0) |
| 17 | 130.30 | 5.61 m | 17ʹ | 130.27 | 5.61 m |
| 18 | 128.17 | 5.67 m | 18ʹ | 128.17 | 5.67 m |
| 19 | 16.63 | 1.73 d (1.0) | 19ʹ | 16.63 | 1.71 d (0.8) |
| 20 | 18.13 | 1.04 s | 20ʹ | 17.97 | 0.99 s |
| 21 | 18.09 | 1.04 s | 21ʹ | 17.94 | 0.99 s |

Red color indicates inaccurate speculation.

## Supplementary Table S8. Selected 50 promoter regions from *B. thailandensis* E264

| **Promoter** | **Gene ID** | **Annotation** | **Length (bp)** | **Gene Expression Quantification (48h)** | |
| --- | --- | --- | --- | --- | --- |
| P1 | BTH_RS00145 | hypothetical protein | 151 | | 46215 |
| P2 | BTH_RS20340 | cold-shock protein | 402 | | 20108 |
| P3 | BTH_RS03745 | hypothetical protein | 964 | | 44536 |
| P4 | BTH_RS26270 | hypothetical protein | 117 | | 6746 |
| P5 | BTH_RS11185 | DUF1843 domain-containing protein | 636 | | 57534 |
| P6 | BTH_RS31200 | ssrS ncRNA | 82 | | 3717 |
| P7 | BTH_RS15255 | phage tail assembly chaperone | 108 | | 15256 |
| P8 | BTH_RS16090 | cold-shock protein | 530 | | 30308 |
| P9 | BTH_RS03210 | 30S ribosomal protein S21 | 78 | | 6569 |
| P10 | BTH_RS08725 | hypothetical protein | 54 | | 9983 |
| P11 | BTH_RS10190 | hypothetical protein | 115 | | 32905 |
| P12 | BTH_RS11175 | DUF1842 domain-containing protein | 52 | | 27643 |
| P13 | BTH_RS10215 | type II toxin-antitoxin system HicB family antitoxin | 43 | | 5112 |
| P14 | BTH_RS24100 | hypothetical protein | 772 | | 8979 |
| P15 | BTH_RS25325 | hypothetical protein | 244 | | 5312 |
| P16 | BTH_RS12210 | DNA-binding protein HU-alpha | 390 | | 3207 |
| P17 | BTH_RS08720 | radical SAM protein | 485 | | 5601 |
| P18 | BTH_RS11180 | DUF1842 domain-containing protein | 87 | | 24170 |
| P19 | BTH_RS13110 | hypothetical protein | 256 | | 3971 |
| P20 | BTH_RS03765 | hypothetical protein | 737 | | 8448 |
| P21 | BTH_RS14795 | hypothetical protein | 276 | | 18382 |
| P22 | BTH_RS27920 | tRNA-Tyr | 485 | | 5657 |
| P23 | BTH_RS14785 | hypothetical protein | 56 | | 13160 |
| P24 | BTH_RS05505 | hypothetical protein | 923 | | 8234 |
| P25 | BTH_RS28255 | H-NS histone | 62 | | 2002 |
| P26 | BTH_RS19265 | DUF883 domain-containing protein | 330 | | 3327 |
| P27 | BTH_RS15950 | 30S ribosomal protein S20 | 207 | | 1405 |
| P28 | BTH_RS09035 | DUF2158 domain-containing protein | 383 | | 1873 |
| P29 | BTH_RS22365 | tRNA-Glu | 150 | | 1047 |
| P30 | BTH_RS25415 | 50S ribosomal protein L35 | 193 | | 6521 |
| P31 | BTH_RS23610 | RNA-binding protein Hfq | 134 | | 2356 |
| P32 | BTH_RS21530 | hypothetical protein | 217 | | 2676 |
| P33 | BTH_RS00265 | hypothetical protein | 100 | | 2566 |
| P34 | BTH_RS18490 | iron-sulfur cluster insertion protein ErpA | 206 | | 1448 |
| P35 | BTH_RS19270 | peroxiredoxin | 330 | | 4663 |
| P36 | BTH_RS13050 | dksA | 54 | | 2106 |
| P37 | BTH_RS13660 | avidin | 252 | | 2441 |
| P38 | BTH_RS27300 | type VI secretion system tube protein Hcp | 72 | | 6548 |
| P39 | BTH_RS25185 | Flp family type IVb pilin | 533 | | 3868 |
| P40 | BTH_RS20950 | 50S ribosomal protein L32 | 90 | | 2056 |
| P41 | BTH_RS27870 | 50S ribosomal protein L7/L12 | 63 | | 1186 |
| P42 | BTH_RS25420 | translation initiation factor IF-3 | 49 | | 2986 |
| P43 | BTH_RS27555 | transposase | 191 | | 2522 |
| P44 | BTH_RS20520 | membrane protein | 251 | | 5210 |
| P45 | BTH_RS08385 | hypothetical protein | 293 | | 16472 |
| P46 | BTH_RS07755 | DUF4148 domain-containing protein | 183 | | 1141 |
| P47 | BTH_RS07750 | CBS domain-containing protein | 176 | | 1998 |
| P48 | BTH_RS19345 | LysR family transcriptional regulator | 560 | | 2622 |
| P49 | BTH_RS04190 | LuxR family transcriptional regulator | 171 | | 1825 |
| P50 | BTH_RS17270 | hypothetical protein | 433 | | 2202 |

## Supplementary Table S9. HRMS data of E264ΔBAC::attB/rhi fermentation products

| peaks | retention time (min) | chemical formula | calculated [M + H] ^+^ | Observed [M + H] ^+^ | Error (ppm) | speculated compounds based on MS |
| --- | --- | --- | --- | --- | --- | --- |
| a | 13.4 | C_35_H_49_NO_9_ | 628.3480 | 628.3483 | -0.4 | Rhizoxin M_1_, Z_1_ or S_2_ |
| b | 14.8 | C_36_H_51_NO_9_ | 642.3637 | 642.3636 | 0.1 | Rhizoxin M_2_ or Z_2_ |
| c | 12.7 | C_35_H_49_NO_9_ | 628.3480 | 628.3479 | 0.2 | Rhizoxin M_1_, Z_1_ or S_2_ |
| d | 13.6 | C_35_H_49_NO_8_ | 612.3531 | 612.3534 | -0.5 | Unsure |
| e | 14.0 | C_35_H_49_NO_9_ | 628.3480 | 628.3479 | 0.2 | Rhizoxin M_1_, Z_1_ or S_2_ |
| f | 14.5 | C_35_H_49_NO_9_ | 628.3480 | 628.3479 | 0.2 | Rhizoxin M_1_, Z_1_ or S_2_ |
| g | 15.3 | C_35_H_47_NO_8_ | 610.3374 | 610.3384 | -1.6 | Unnamed rhizoxins*^a^* |
|  | 15.3 | C_36_H_51_NO_9_ | 642.3637 | 642.3645 | -1.4 | Rhizoxin M_2_ or Z_2_ |
| h | 15.7 | C_35_H_47_NO_9_ | 626.3687 | 626.3690 | -0.6 | Unnamed rhizoxins*^b^* |


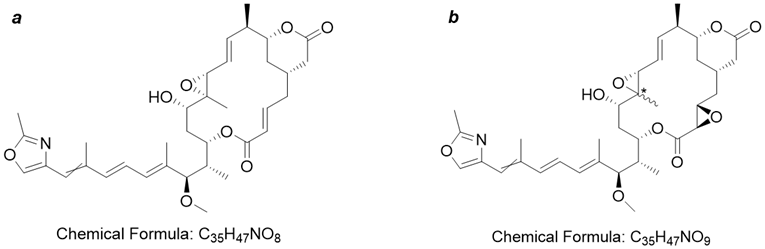


# Supplementary Figures


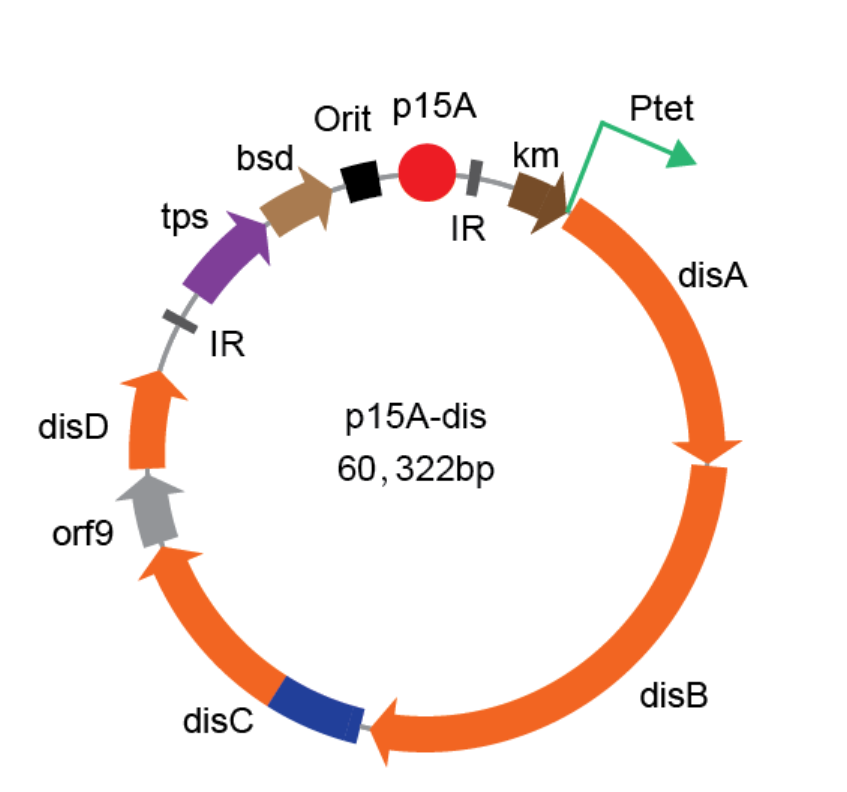


## Supplementary Figure S1. Plasmid map of p15A-dis.


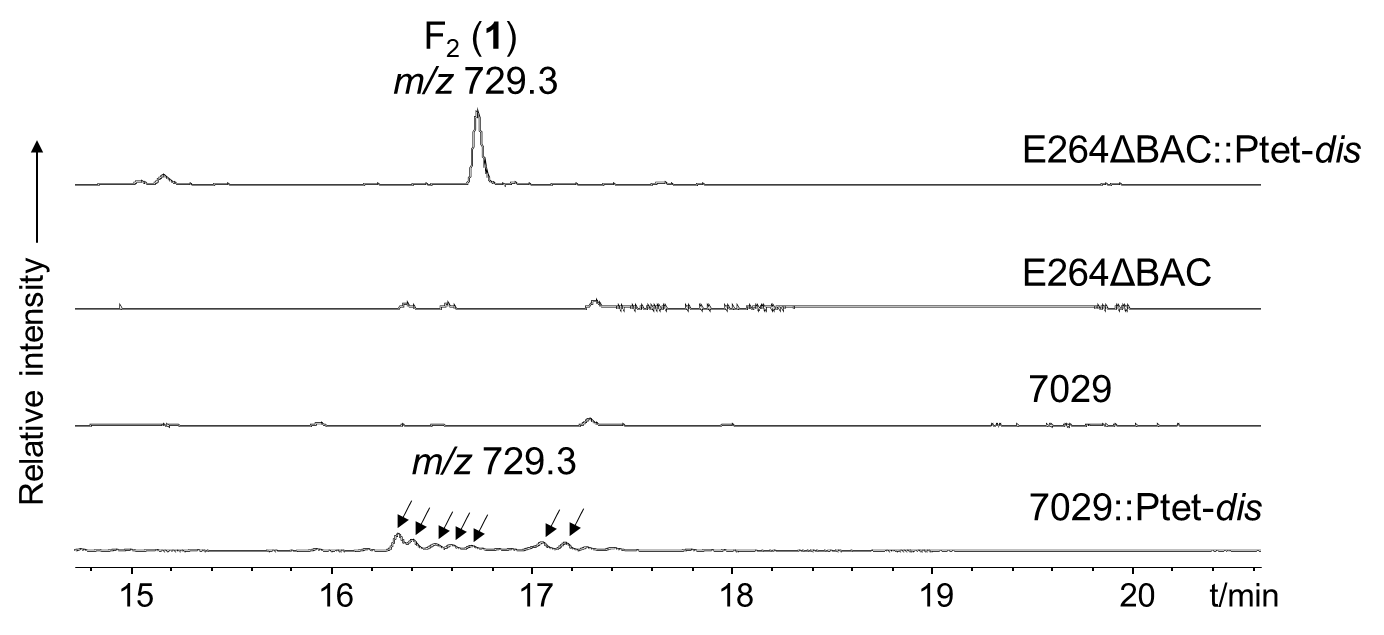


## Supplementary Figure S2. Heterologous expression of disorazol gene cluster in different hosts.

## Supplementary Figure S3. The ^1^H NMR spectrum of disorazol F_2_ (1) in MeOH-*d*_4_

## Supplementary Figure S4. The ^13^C NMR spectrum of disorazol F_2_ (1) in MeOH-*d*_4_


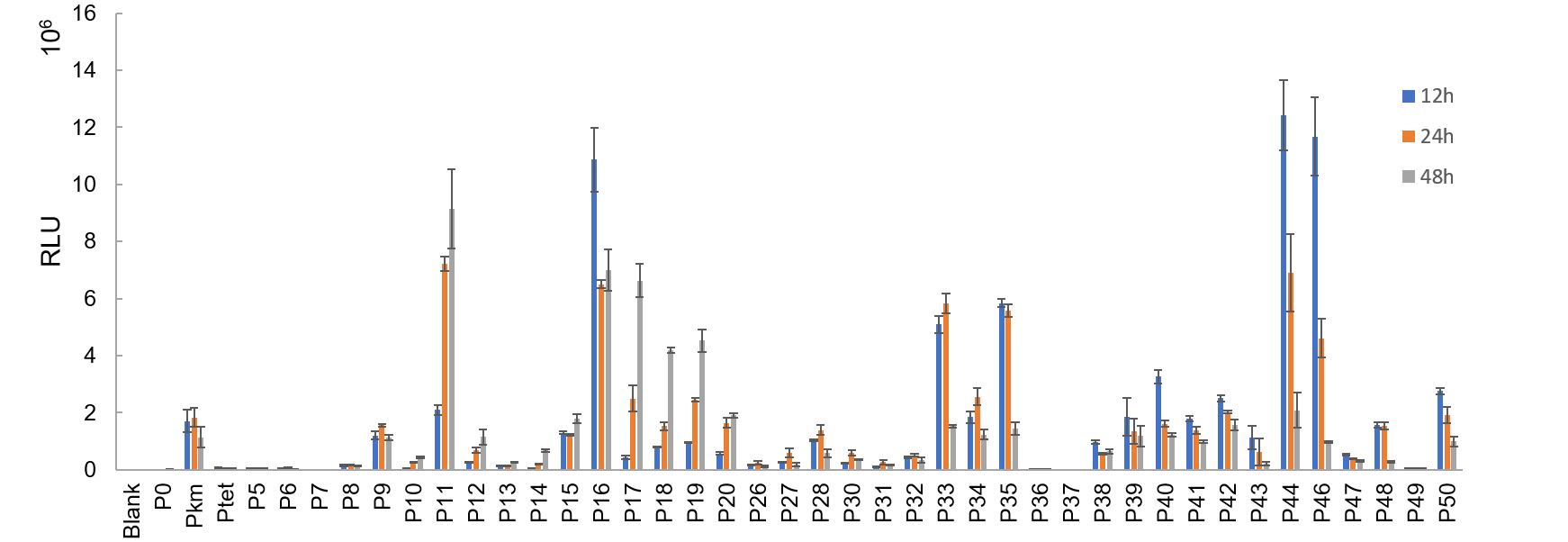


## Supplementary Figure S5. The promoter strength evaluated by firefly luciferase assay. The error bars were calculated based on three independent biological replicates using standard deviation.

**
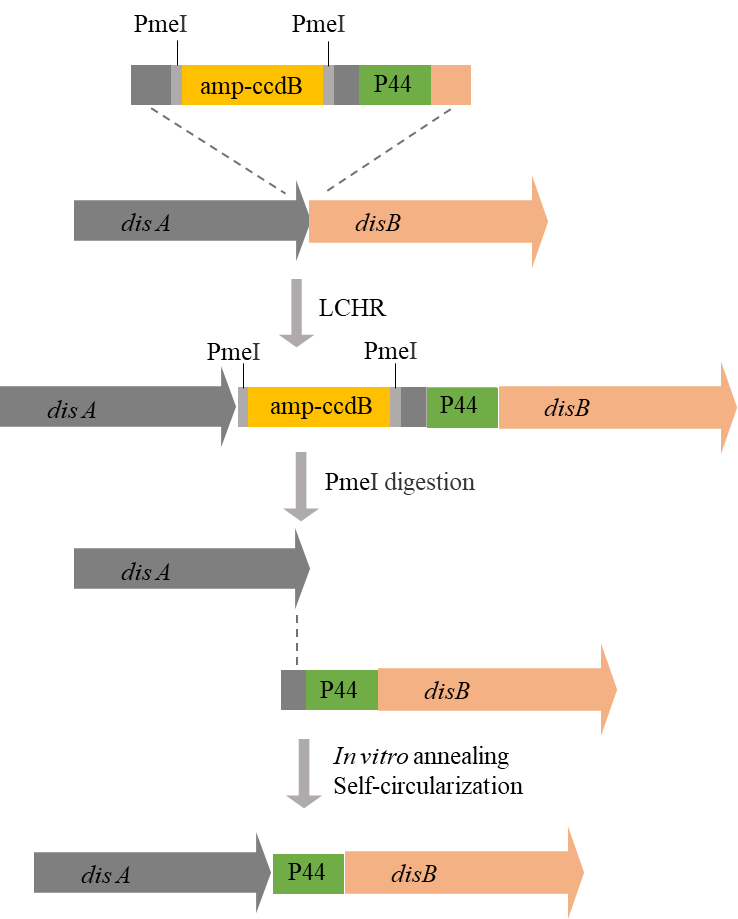
**

## Supplementary Figure S6. Seamless insertion of promoter P44.

##
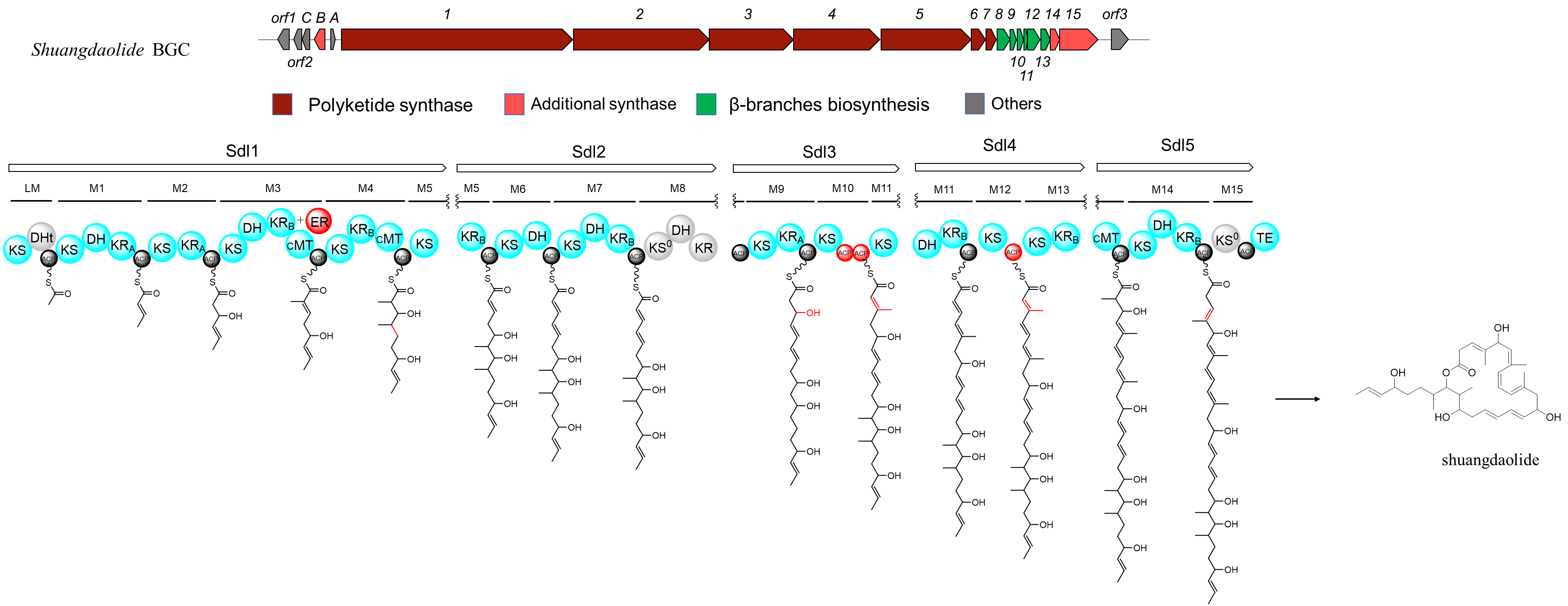
**Supplementary Figure S7.** Proposed biosynthesis of shuangdaolides.

**References**

1. Q. Ouyang *et al.*, Promoter screening facilitates heterologous production of complex secondary metabolites in Burkholderiales Strains. *ACS Synth. Biol.* **9**, 457-460 (2020).

2. H. Wang *et al.*, ExoCET: exonuclease in vitro assembly combined with RecET recombination for highly efficient direct DNA cloning from complex genomes. *Nucleic Acids Res.* **46**, e28 (2017).

3. C. Dehio, M. Meyer, Maintenance of broad-host-range incompatibility group P and group Q plasmids and transposition of Tn5 in *Bartonella henselae* following conjugal plasmid transfer from *Escherichia coli*. *J. Bacteriol.* **179**, 538-540 (1997).

4. P. J. Brett, D. Deshazer, D. E. Woods, *Burkholderia thailandensis* sp. nov., a *Burkholderia pseudomallei*-like species. *Int. J. Syst. Evol. Microbiol.* **48**, 317-320 (1998).
